# Supplementary material for: Fan cells in lateral entorhinal cortex directly influence medial entorhinal cortex through synaptic connections in layer 1
Source: eLife. 2022 Dec 23;11:e83008. doi: 10.7554/eLife.83008 (PMC9822265; doi:10.7554/eLife.83008)
Supplement: Figure 4—source data 1. [file elife-83008-fig4-data1.docx]

| **Neuron type** | **VM** | **IR+** | **IR-** | **Sag** | **AP Threshold** | **AP Half-Width** | **Rheobase** | **Resonance frequency** |
| --- | --- | --- | --- | --- | --- | --- | --- | --- |
| L1 IN | -68.6 ± 1.4 | 182.2 ± 42.3 | 184.6 ± 23.8 | -0.96 ± 1.9 | -32.8 ​​± 1.3 | 0.66 ± 0.1 | 178.4 ± 19.3 | 2.1 ± 0.35 |
| L2 SC | -65.6 ±  0.42 | 80.34 ± 5.7 | 66.0 ±  4.1 | 0.64 ±  0.01 | -40.4 ±  0.45 | 0.50 ±  0.7 | 80.6 ±  74.2 | 8.15 ±  2.9 |
| L2 PC | -68.5 ± 0.79 | 135.7 ±  13.9 | 109.8 ±  8.8 | 0.77 ± 0.2 | -39.5 ± 1.2 | 0.52 ± 0.01 | 131.4 ± 15.1 | 3.40 ± 0.40 |
| L2 IN | -70.6 ± 1.1 | 132.4 ± 17.7 | 123.9 ± 16.5 | 0.86 ± 0.01 | -37.0 ± 1.0 | 0.26 ± 0.03 | 269.2 ± 34.9 | 3.2 ± 0.82 |
| L3 PC | -70.6 ± 1.4 | 212.5 ± 14.2 | 142.8 ± 14.2 | 0.83 ± 0.02 | -40.2 ± 1.2 | 0.57 ± 0.02 | 84.9 ± 7.4 | 1.8 ± 0.28 |
| L5a PC | -64.2 ± 2.4 | 270.9 ± 23.3 | 222.5 ± 19.4 | 0.85 ± 0.03 | -35.4 ± 1.3 | 0.69 ± 0.04 | 57.8 ± 9.4 | 1.8 ± 0.28 |
| L5b PC | -74.3 ± 2.0 | 341.4 ± 66.1 | 218.0 ± 31.5 | 0.82 ± 0.02 | -39.3 ± 5.1 | 0.71 ± 0.6 | 73.9 ± 18.6 | 1.8 ± 0.02 |

**Figure 4 – source data 1: Summary of key subthreshold and suprathreshold properties for each type of neuron recorded in the medial entorhinal cortex**

Data is shown for stellate cells (SC), pyramidal cells (PC) and interneurons (IN) in layers (L) 1, 2, 3, 5a, and 5b of medial entorhinal cortex. Values are mean and standard error of the mean. Abbreviations: VM, resting membrane potential (volts); IR, input resistance;  AP, action potential.
