# Supplementary material for: Fan cells in lateral entorhinal cortex directly influence medial entorhinal cortex through synaptic connections in layer 1
Source: eLife. 2022 Dec 23;11:e83008. doi: 10.7554/eLife.83008 (PMC9822265; doi:10.7554/eLife.83008)
Supplement: Figure 4—source data 2. [file elife-83008-fig4-data2.docx]

| **Comparison** | **Z-statistic** | ***p-*value, unadjusted** | ***p*-value, adjusted** |
| --- | --- | --- | --- |
| L2 SC-L2 PC | 3.889 | < 0.001*** | 0.001** |
| L2 SC-L3 PC | 0.266 | 0.790 | 1 |
| L2 SC-L5a PC | -0.969 | 0.333 | 1 |
| L2 SC-L5b PC | 0.576 | 0.565 | 1 |
| L2 PC-L3 PC | 2.561 | 0.010* | 0.104 |
| L2 PC-L5a PC | 0.109 | 0.310 | 1 |
| L2 PC-L5b PC | 1.333 | 0.183 | 1 |
| L3 PC-L5a PC | -1.014 | 0.310 | 1 |
| L3 PC-L5b PC | 0.478 | 0.633 | 1 |
| L5a PC-L5b PC | 1.039 | 0.299 | 1 |

**Figure 4 – source data 2 Summary of pairwise comparisons of membrane potential responses for different types of principal cell in medial entorhinal cortex**

Posthoc pairwise comparisons of excitatory postsynaptic membrane potential amplitude were made using a Dunn test and *p-*values were adjusted with a Bonferroni correction for multiple comparisons. Responses were found to be significantly larger in layer 2 (L2) pyramidal cells (PCs) compared to layer 2 stellate cells (SCs), with no other significant difference between cell types. Data was compared for principal cells in layers (L) 2-5b. Asterisks indicate significance (*p* < 0.05*, *p* < 0.01**, *p* < 0.001***)
