# Supplementary material for: Fan cells in lateral entorhinal cortex directly influence medial entorhinal cortex through synaptic connections in layer 1
Source: eLife. 2022 Dec 23;11:e83008. doi: 10.7554/eLife.83008 (PMC9822265; doi:10.7554/eLife.83008)
Supplement: Figure 6—source data 1. [file elife-83008-fig6-data1.docx]

| **Cell type** | **GABA antagonist order** | **EPSP amplitude: n (p < 0.01)** | **EPSP halfwidth: n (p < 0.01)** | **IPSP amplitude: n (p < 0.01)** |
| --- | --- | --- | --- | --- |
| L2 SC | Con -> GZ -> GZ + CGP | 5/5 (100%) | 3/3 (100.0%) | 3/5 (60.0%) |
| L2 PC | Con -> GZ -> GZ + CGP | 3/5 (60.0%) | 4/4 (100.0%) | 5/5 (100.0%) |
| L2 SC | Con -> CGP -> GZ + CGP | 2/4 (50.0%) | NA | 4/4 (100.0%) |
| L2 PC | Con -> CGP -> GZ + CGP | 0/1 (0.0%) | 1/1 (100.0%) | 1/1 (100.0%) |

**Figure 6 – source data 1: Summary of cell-by-cell analysis of membrane potential responses after application of GABA receptor antagonists.**

Data for each cell was analysed using a Friedman test where the dependent variable was the membrane potential response, the within-subjects factor was GABA receptor antagonist (Gabazine (GZ) or CGP55485 (CGP)), and responses were grouped by sweep of optical stimulus (30-50 sweeps per cell). Table shows the proportion of layer 2 stellate (SC) and pyramidal cells (PC) where there was a significant effect (p < 0.01) of drug on EPSP amplitude, EPSP halfwidth or IPSP amplitude. For analysis of EPSP halfwidth, neurons with average responses < 1 mV were excluded to ensure accuracy of measurements at the level of a single pulse. Data is shown for neurons where Gabazine was applied before CGP55485 (top two rows) and vice versa (bottom two rows).
