# Supplementary material for: Fan cells in lateral entorhinal cortex directly influence medial entorhinal cortex through synaptic connections in layer 1
Source: eLife. 2022 Dec 23;11:e83008. doi: 10.7554/eLife.83008 (PMC9822265; doi:10.7554/eLife.83008)
Supplement: Figure 6—source data 3. [file elife-83008-fig6-data3.docx]

| **Response, cell type** | **GABA antagonist order** | **Con vs CGP (p < 0.01)** | **Con vs GZ (p < 0.01)** | **GZ vs CGP (p < 0.01)** |
| --- | --- | --- | --- | --- |
| EPSP amplitude,  L2 SC | Con -> CGP -> GZ + CGP | 1/2 (50.0%) | 1/2 (50.0%) | 1/2 (50.0%) |
| EPSP halfwidth,  L2 SC | Con -> CGP -> GZ + CGP | NA | NA | NA |
| IPSP amplitude,  L2 SC | Con -> CGP -> GZ + CGP | 3/4 (75.0%) | 3/4 (75.0%) | 3/4 (75.0%) |
| EPSP amplitude,  L2 PC | Con -> CGP -> GZ + CGP | NA | NA | NA |
| EPSP halfwidth,  L2 PC | Con -> CGP -> GZ + CGP | 0/1 (0.0%) | 0/1 (0.0%) | 0/1 (0.0%) |
| IPSP amplitude,  L2 PC | Con -> CGP -> GZ + CGP | 1/1 (100.0%) | 1/1 (100.0%) | 0/1 (0.0%) |

**Figure 6 – source data 3: Summary of posthoc pairwise comparisons of membrane potential responses after application of GABA receptor antagonists**

Posthoc pairwise comparisons were performed for each cell for each membrane response characteristic where a Friedman test revealed a significant effect of GABA receptor antagonists (cf. Figure 6, Supplement 1). Data is only shown for neurons with a significant result from a Friedman test. Responses were grouped by sweep of optical stimulus (30-50 sweeps per cell) and compared across conditions using a paired Wilcoxon rank sum test. All possible comparisons were included; control (Con) vs CGP55484 (CGP), control vs Gabazine (Gabazine) + CGP55485, and CGP55485 vs Gabazine + CGP55485. Table shows the proportion of layer 2 stellate (SC) and pyramidal cells (PC) where there was a significant effect (p < 0.01) of drug on EPSP amplitude, EPSP halfwidth, or IPSP amplitude after adjustment of p-values for multiple comparisons. Data is shown for neurons where CGP55484 was applied before Gabazine.
